# Exploration of potential shared gene signatures between periodontitis and multiple sclerosis

**DOI:** 10.1186/s12903-023-03846-7

**Published:** 2024-01-13

**Authors:** Erli Wu, Ming Cheng, Xinjing Zhang, Tiangang Wu, Shuyan Sheng, Mengfei Sheng, Ling Wei, Lei Zhang, Wei Shao

**Affiliations:** 1 College & Hospital of Stomatology, Key Lab. of Oral Diseases Research of Anhui Province, Anhui Medical University, Hefei, 230032 China; 2 First Clinical Medical College (First Affiliated Hospital), Anhui Medical University, Hefei, 230032 China; 3 Department of Microbiology and Parasitology, Anhui Provincial Laboratory of Pathogen Biology, School of Basic Medical Sciences, Anhui Medical University, Hefei, Anhui China; 4 The First Affiliated Hospital of Anhui Medical University, Hefei, Anhui 230022 China; 5 Department of Periodontology, Anhui Stomatology Hospital affiliated to Anhui Medical University, Hefei, 230032 China

**Keywords:** Periodontitis, Multiple sclerosis, Transcriptomic analysis, Crosstalk genes, Immune infiltration, Biomarker

## Abstract

**Background:**

Although periodontitis has previously been reported to be linked with multiple sclerosis (MS), but the molecular mechanisms and pathological interactions between the two remain unclear. This study aims to explore potential crosstalk genes and pathways between periodontitis and MS.

**Methods:**

Periodontitis and MS data were obtained from the Gene Expression Omnibus (GEO) database. Shared genes were identified by differential expression analysis and weighted gene co-expression network analysis (WGCNA). Then, enrichment analysis for the shared genes was carried out by multiple methods. The least absolute shrinkage and selection operator (LASSO) regression was used to obtain potential shared diagnostic genes. Furthermore, the expression profile of 28 immune cells in periodontitis and MS was examined using single-sample GSEA (ssGSEA). Finally, real-time quantitative fluorescent PCR (qRT-PCR) and immune histochemical staining were employed to validate Hub gene expressions in periodontitis and MS samples.

**Results:**

*FAM46C*, *SLC7A7*, *LY96*, *CFI*, *DDIT4L*, *CD14*, *C5AR1*, and *IGJ* genes were the shared genes between periodontitis, and MS. GO analysis revealed that the shared genes exhibited the greatest enrichment in response to molecules of bacterial origin. LASSO analysis indicated that *CFI*, *DDIT4L*, and *FAM46C* were the most effective shared diagnostic biomarkers for periodontitis and MS, which were further validated by qPCR and immunohistochemical staining. ssGSEA analysis revealed that T and B cells significantly influence the development of MS and periodontitis.

**Conclusions:**

*FAM46C*, *SLC7A7*, *LY96*, *CFI*, *DDIT4L*, *CD14*, *C5AR1*, and *IGJ* were the most important crosstalk genes between periodontitis, and MS. Further studies found that *CFI*, *DDIT4L*, and *FAM46C* were potential biomarkers in periodontitis and MS.

**Supplementary Information:**

The online version contains supplementary material available at 10.1186/s12903-023-03846-7.

## Introduction

Periodontitis is a common chronic infectious and inflammatory disease affecting people worldwide. Its etiology mainly includes the direct damage of the periodontal tissues by bacteria and the immune disorder of the host caused by bacteria [1]. Periodontitis is distinguished by enduring inflammation of the tissues that support the teeth, destruction of the periodontal ligaments, and progressive loss of alveolar bone around the teeth [2]. Recently, it has been shown that periodontitis can lead to several systemic illnesses. This may be due to the pro-inflammatory cytokines or bacteria in the mouth through the blood or triggering the body’s immune response and other related mechanisms [3].

Multiple sclerosis (MS) is an autoimmune disease that causes inflammatory demyelinating lesions of white matter in the central nervous system [4]. Even though the cause of MS is unclear, current findings suggest that environmental and genetic variables contribute to the disease’s development [5]. Several environmental factors, such as infection, latitude, vitamin D deficiency, and smoking, contribute to the development of MS [6]. Research has shown that bacterial infection may be a crucial factor in the etiology of MS. They were found to be pathogenic environmental factors in the pathogenesis of MS [13]. In addition, some pathogenic or symbiotic bacteria can mediate MS by activating Th17 cells to produce inflammatory factors. Studies have shown that *Porphyromonas gingivalis (P. gingivalis)* is significantly elevated in patients with MS, and *P. gingivalis* is also one of the main causative agents of periodontitis [7]. Also, people with periodontitis are more susceptible to MS, and periodontal infections may worsen MS symptoms [8]. These findings suggest that there could be links between periodontitis and MS. However, the molecular mechanisms and pathological interactions between the two remain unclear.

As microarray and high-throughput sequencing technologies continue to advance quickly, bioinformatics techniques are frequently used to investigate the crosstalk between diseases in order to reveal the connections between the cellular and molecular mechanisms of diseases. In this study, we explored potential crosstalk genes between periodontitis and MS through bioinformatics methods. We analyzed the interactions between these genes and immune cells to acquire a greater comprehension of potential mechanisms of interaction between periodontitis and MS. Additionally, three candidate biomarkers for periodontitis and MS were identified by using bioinformatics tools, which were further validated by qPCR and immunohistochemical staining techniques, suggesting that they may be biomarkers for predicting the occurrence of periodontitis and MS.

## Materials and methods

### Data download

Gene expression data for periodontitis and MS were downloaded from the Gene Expression Omnibus (GEO) database (https://www.ncbi.nlm.nih.gov/geo/). In the periodontitis dataset, GSE16134 (based on the GPL570 platform) was used as a test cohort with 310 gingival papillae (241 “diseased” and 69 “healthy”), and GSE1334 as a validation cohort with 247 gingival papillae with 183 “diseased” and 64 “healthy.” The MS dataset contains GSE108000 (based on the GPL13497 platform) and GSE135511 (based on the GPL6883 platform), and we combined GSE108000 and GSE135511 into a new dataset by using the “SVA” R package to remove batches. The combined dataset includes 20 healthy controls and 70 MS samples. In addition, to assess the effectiveness of the diagnostic process, we downloaded the GSE38010 dataset (based on the GPL570 platform), which contains 2 healthy controls and 5 MS samples.

### Identification of DEGs

To normalize the datasets, R (4.2.3) software was used. Afterward, we identified differentially expressed genes (DEGs) from the GSE16134 and a combined dataset of the GSE108000 and GSE135511 by using the R package “limma” with adjusted *P* values < 0.05 and |log FC|≥0.8.

### WGCNA network construction and module identification

The co-expression network of periodontitis (GSE16134) and MS (a merged dataset of GSE108000 and GSE135511) was constructed using the WGCNA package in R. The network is ensured to be a scale-free network by using a soft threshold, which is advantageous for subsequent network generation. Gene modules were identified using hierarchical clustering trees, while gene modules with strong connections were constructed using hierarchical clustering based on topological overlap matrix (TOM). Pearson’s correlation coefficient was calculated to analyze relationships between the various modules and diseases. The module showing the highest correlation with the disease was selected, and the genes within this module were obtained.

### Identification of shared genes and pathway enrichment

By drawing Venn diagrams, the shared genes identified by WGCNA and DEG were obtained. Then, we explored functions and pathways associated with these genes through Gene Ontology (GO) and Kyoto Encyclopedia of Genes and Genomes (KEGG) using “clusterProfiler” and “org.Hs.eg.db” packages [9–12].

### Feature selection by the least absolute shrinkage and selection operator

To discover hub genes with the best diagnostic efficacy among the shared genes identified above between periodontitis and MS, we utilized the “glmnet” package in R to conduct the least absolute shrinkage and selection operator (LASSO) regression.

### Candidate biomarker expression levels and diagnostic value

We utilized the “ggplot2” package in R software to test expression levels of the hub genes in periodontitis and MS samples. To assess the diagnostic efficacy of potential biomarkers on periodontitis (GSE16134) and MS (a merged dataset of GSE108000 and GSE135511) datasets, we used receiver operating characteristic curves (ROCs) using the “pROC” package in R. Furthermore, we verify the diagnostic efficiency of potential biomarkers using two external datasets including GSE10334 and GSE38010.

#### ssGSEA

We analyzed the infiltration of immune cells in diseased and healthy samples through ssGSEA using the “GSVA” R package. Then, we explored links between potential biomarkers and infiltrating immune cells through the Spearman method.

### Gingival biopsy and peripheral blood collection

10 human gingival tissues, including 5 cases and 5 controls, were obtained from healthy volunteers and patients with periodontitis. In addition, our study also included individuals with 5 MS samples and 10 healthy volunteers, and we obtained peripheral blood from multiple sclerosis patients and healthy people, respectively, for the extraction of peripheral blood mononuclear cells (PBMCs). Inclusion criteria included patients diagnosed and treated for the first time, patients with complete medical records, and patients without systemic disorders. All studies were approved by the Ethics Committee of the Affiliated Stomatology Hospital of Anhui Medical University and the First Affiliated Hospital of Anhui Medical University.

### RNA collection and qRT-PCR

A Ficoll (Histopaque; Sigma–Aldrich, Zwijndrecht, The Netherlands) density gradient was used to extract PBMCs through centrifugation. RNA from gingival tissue and PBMCs was extracted using TRIzol reagent (Invitrogen). cDNAs was synthesized from 2 µg total RNA according to instructions of cDNA Reverse Transcription Kit (Takara, Tokyo, Japan). Subsequently, qRT-PCR was performed using the Stratagene Mx3000P system (Agilent Technologies, USA) and SYBR Green Master Mix (11,701, Accurate Biology). GAPDH was used to normalize the gene’s expression levels, and the comparative Ct method with Formula ^2−ΔCt^ was used to compute the expression value. All experiments were repeated more than three times. Supplementary Table S1 contains a list of primers.

### Immunohistochemical staining of gingival tissue

The collected gingival tissues were preserved using 4% paraformaldehyde and then embedded in paraffin. The paraffin-embedded tissue was sliced into serial Sect. 4 micrometers thick and then deparaffinized for antigen extraction. Subsequently, these slides were treated with goat serum and then incubated with antibodies. After that, 3,3’-diaminobenzidine tetrahydrochloride (DAB) and hematoxylin were used to stain the sections. Microscope images were captured and processed using image-processing software (ImageJ v 1.48).

### Statistical analysis

We utilized GraphPad Prism 8.0 for both conducting statistical analysis and creating visual representations. All results are expressed as mean ± standard deviation. The method chosen for statistical analysis was the unpaired t-test (*P* < 0.05).

## Results

### Identification of DEGs

**Fig. 1:**
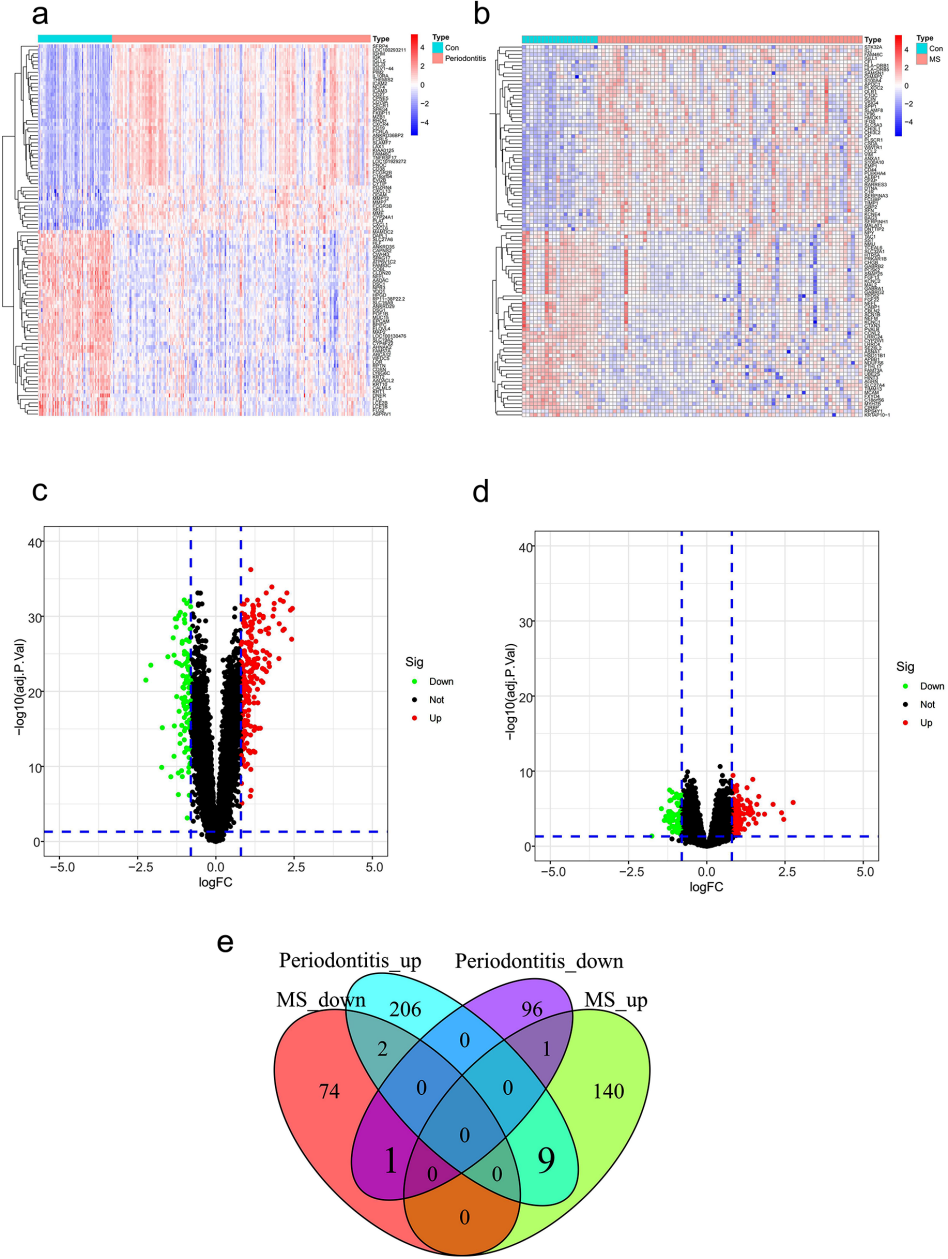
identification of genes with variable expression. The periodontitis database GSE16134’s top 100 DEGs are depicted in a heatmap in Figure **(a)**. **(b)** In a combined dataset of GSE108000 and GSE135511 in MS, a heatmap of the top 100 DEGs. **(c)** A DEG volcano graphic from the GSE16134 periodontitis database. **(d)** A volcano plot of DEGs in the MS dataset created by merging GSE108000 and GSE135511. **(e)** A Venn diagram showing an overlap of 10 DEGs between periodontitis and MS. Control is a negative; MS is multiple sclerosis. Differentially expressed genes, or DEGs

In GSE16134, a total of 315 DEGs with 217 upregulated and 98 downregulated, were found, while the combined dataset of GSE108000 and GSE135511 showed 227 DEGs, 150 of which were upregulated and 77 downregulated. The top 100 DEGs of these two diseases were shown in heatmaps (Fig. 1a, b), and expression patterns of the DEGs in these diseases were displayed in volcano maps (Fig. 1c, d). Ten genes (*FAM46C, COL4A1, SLC7A7, LY96, CFI, DDIT4L, CD14, C5AR1, IGJ, NEFL*) differently expressed in both MS and periodontitis were revealed by combining the upregulated and downregulated genes (Fig. 1e).

### WGCNA network construction and module identification

**Fig. 2:**
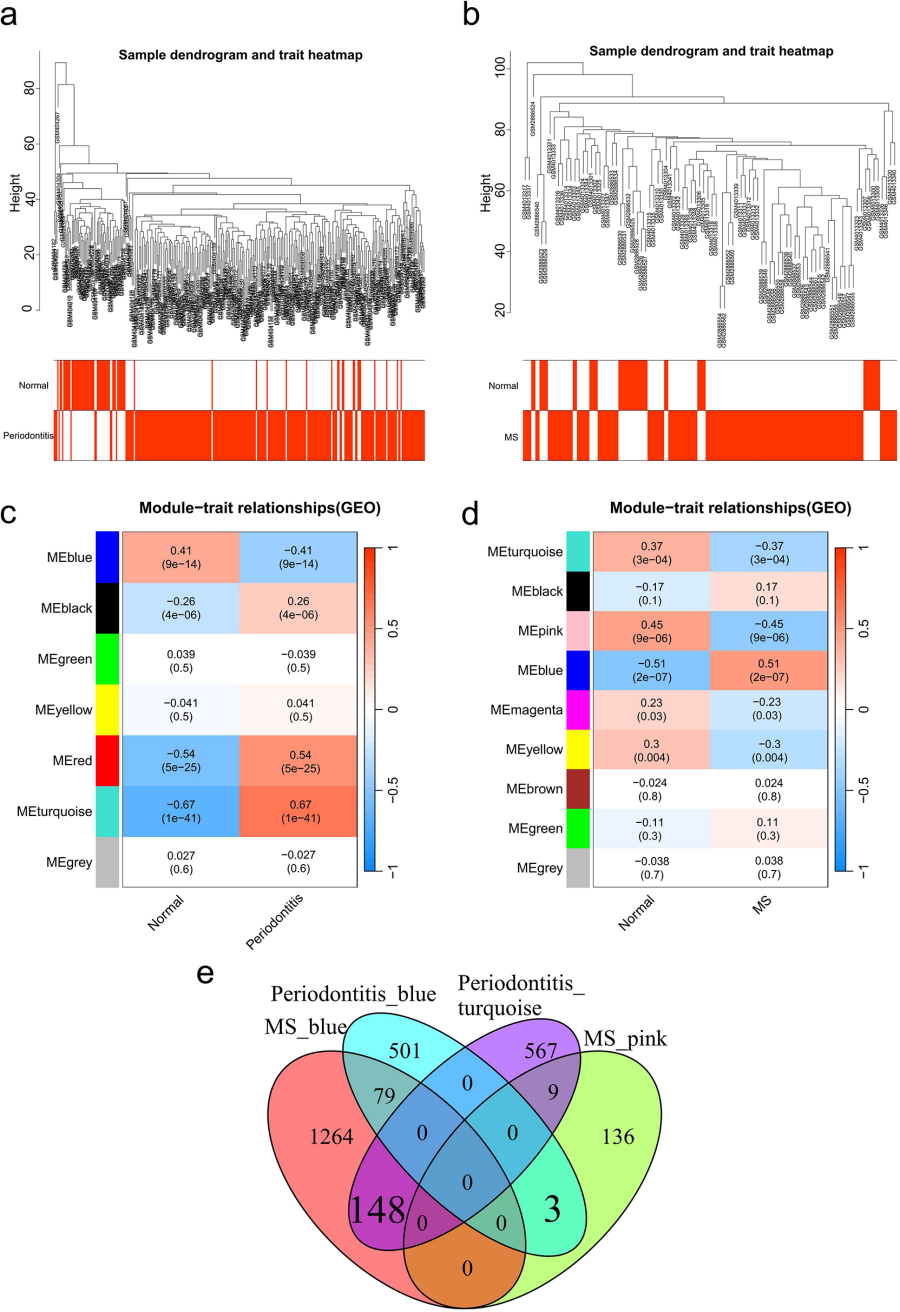
Analysis of coexpression for genes with differential expression. **(a)** Sample dendrogram and trait heatmap in the periodontitis database GSE16134. **(b)** Sample dendrogram and trait heatmap in a merged dataset of GSE108000 and GSE135511 in MS. **(c)** Heatmap of the module-trait relationships in the periodontitis database GSE16134. **(d)** Heatmap of the module-trait connections in the combined GSE108000 and GSE135511 dataset in MS. **(e)** Venn diagram shows that 151 genes overlap in MS and periodontitis modules. MS: Multiple sclerosis

By clustering samples to check the outliers, neither GSE16134 nor a combined dataset of GSE108000 and GSE135511 deleted the samples (Fig. 2a, b). To ensure the creation of a scale-free network, a power of β = 12 was used for GSE16134, while the β value was 3 for the combined GSE108000 and GSE135511 datasets. The co-expression network generated by periodontitis samples consisted of 7 modules, whereas the network constructed using MS samples contained 9 modules (Fig. 2c, d). The Pearson correlation coefficient was applied to calculate the associations of modules with disease. In GSE16134, the turquoise module had the largest positive correlation with periodontitis (*r* = 0.67), while the blue module showed the most significant negative correlation (*r* = -0.41). In a combined dataset of GSE108000 and GSE135511, the blue module had the largest positive association for MS (*r* = 0.51), whereas the pink module had the most significant negative correlation (*r* = -0.45). There were 151 overlapping genes obtained by intersecting genes in the most obvious positive correlation and negative correlation modules (Fig. 2e).

### Identification of shared genes and pathway enrichment

**Fig. 3:**
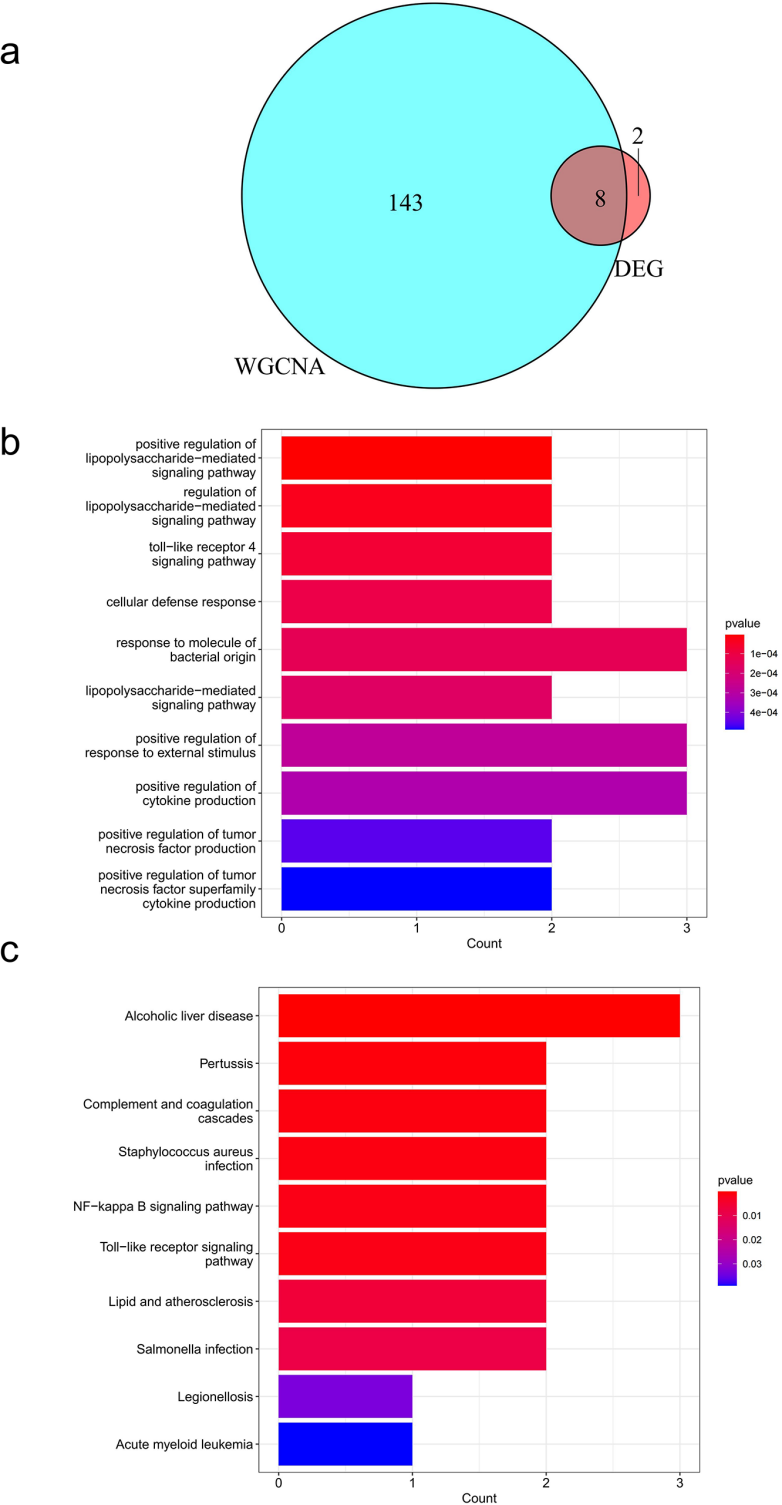
Identification of the shared genes and their KEGG pathway analysis and GO functional enrichment analysis. **(a)** Venn diagram showing that 8 genes were elected from the union set between DEGs and trait-module key genes in WGCNA. **(b)** GO analysis of the shared genes. **(c)** KEGG pathway enrichment analysis of the shared genes. DEG: differentially expressed gene; WGCNA: weighted gene co-expression network analysis

Venn diagrams revealed that there were eight shared genes (*FAM46C*, *SLC7A7*, *LY96*, *CFI*, *DDIT4L*, *CD14*, *C5AR1*, and *IGJ*) that overlapped between periodontitis and MS which were screened by WGCNA and DEGs (Fig. 3a). The GO analysis indicated that these shared genes were most significantly associated with response to molecule of bacterial origin, positive regulation of response to external stimulus, and positive regulation of cytokine production (Fig. 3b). According to the KEGG analysis, these genes were primarily enriched in alcoholic liver disease (ALD), pertussis, complement and coagulation cascades, staphylococcus aureus infection, NF-κB signaling pathway, Toll-like receptor signaling pathway, lipid and atherosclerosis, and salmonella infection (Fig. 3c).

### Identification of potential shared diagnostic genes by least absolute shrinkage and selection operator

**Fig. 4:**
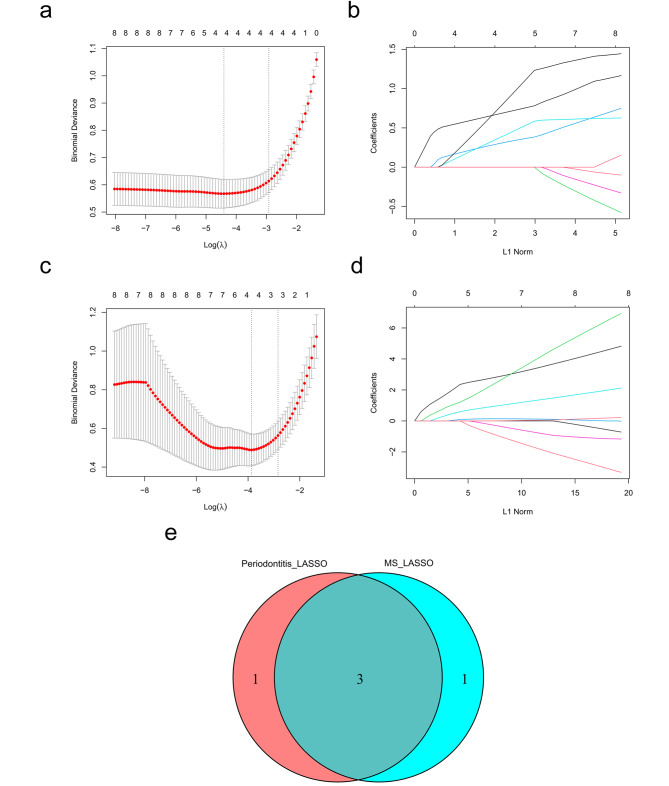
Identification of potential shared diagnostic genes by the LASSO regression model. **(a)** Tenfold cross-validation to select the optimal tuning parameter log (lambda) in the the periodontitis database GSE16134 database. **(b)** LASSO coefficient profiles of diagnostic genes in the the periodontitis database GSE16134 database. **(c)** Tenfold cross-validation to select the optimal tuning parameter log (lambda) in a merged dataset of GSE108000 and GSE135511 in MS. **(d)** LASSO coefficient profiles of diagnostic genes in a merged dataset of GSE108000 and GSE135511 in MS. **(e)** Venn diagram showing the optimal diagnostic biomarkers

A LASSO regression method was utilized to identify the diagnostic gene common to both disorders. Four core cross-genes were found in the periodontitis dataset GSE16134 (Fig. 4a, b), and four core cross-genes were found in the MS dataset merged in GSE108000 and GSE135511 (Fig. 4c, d). Three overlapping genes (*FAM46C*, *CFI*, and *DDIT4L*) were identified as the most effective diagnostic biomarkers for both periodontitis and MS by using a Venn diagram (Fig. 4e).

### Candidate biomarker expression levels and diagnostic value

**Fig. 5:**
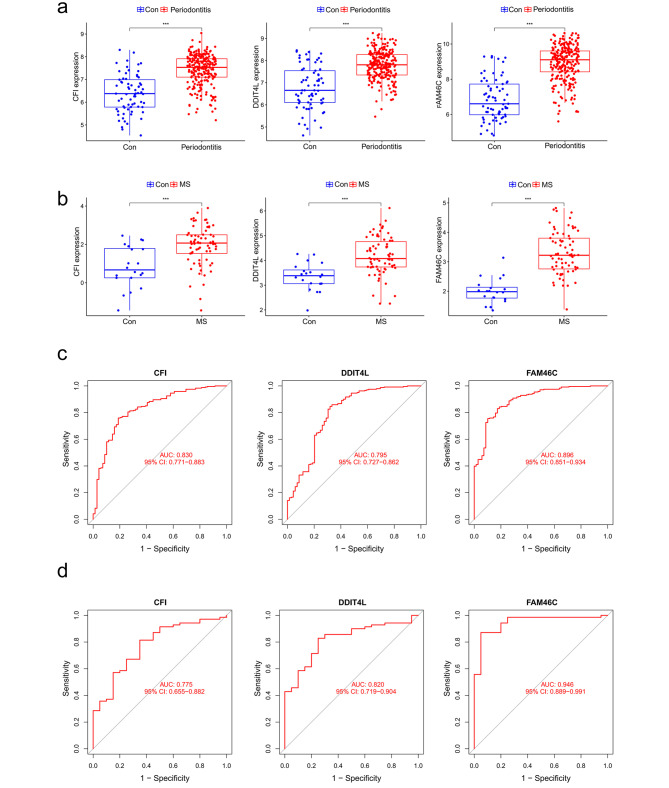
Expression pattern validation and diagnostic value. **(a)** Expression of *CFI*, *DDIT4L* and *FAM46C* in the periodontitis database GSE16134. **(b)** Expression of *CFI*, *DDIT4L* and *FAM46C* in a merged dataset of GSE108000 and GSE135511 in MS. **(c)** ROC curve of the shared diagnostic genes in the periodontitis database GSE16134. **(d)** ROC curve of the shared diagnostic genes in a merged dataset of GSE108000 and GSE135511 in MS. Con: control; MS: Multiple sclerosis. *P < 0.05; **P < 0.01; ***P < 0.001

Further studies found that three candidate biomarkers (*FAM46C*, *CFI*, and *DDIT4L*) expression levels were all upregulated in both periodontitis and MS samples (Fig. 5a, b). ROC curves were employed to evaluate the diagnostic efficacy of these potential biomarkers. In GSE16134 (Fig. 5c), the diagnostic value of these three biomarkers was high: *FAM46C* (AUC = 0.896), *CFI* (AUC = 0.830), and *DDIT4L* (AUC = 0.795). In a dataset merged from GSE108000 and GSE135511 (Fig. 5d), *CFI* (AUC = 0.775) and *DDIT4L* (AUC = 0.820) exhibited greater diagnostic utility for MS, while *FAM46C* demonstrated an almost flawless diagnostic value (AUC = 0.946). Then, two external datasets (GSE10034 and GSE38010) were further used to verify the prediction accuracy of *CFI*, *DDIT4L*, and *FAM46C*. All three showed strong predictive performance (Supplementary Fig. S1).

### Immune infiltration analysis

**Fig. 6:**
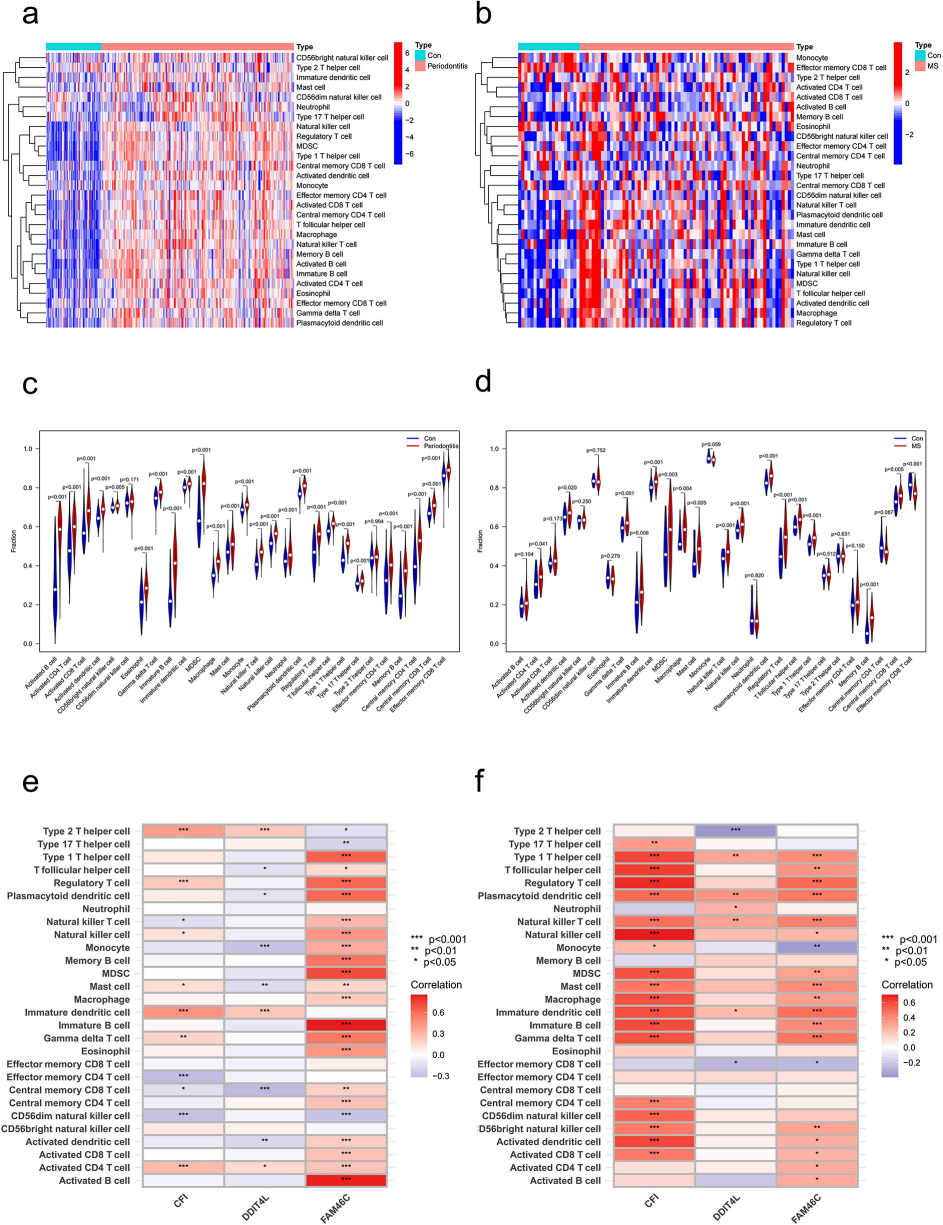
Analysis of immune infiltration associated with periodontitis and MS. **(a)** A heatmap of the distribution of 28 immune cells in normal samples and periodontitis samples. **(b)** A heatmap of the distribution of 28 immune cells in normal samples and MS samples. **(c)** A violin plot of the distribution of 28 immune cells in normal samples and periodontitis samples. **(d)** A violin plot of the distribution of 28 immune cells in normal samples and MS samples. **(e)** The relationship between diagnostic genes and immune cell infiltration in the periodontitis dataset GSE16134. **(f)** The relationship between diagnostic genes and immune cell infiltration in the MS dataset merged by GSE108000 and GSE135511. Con, control; MS: Multiple sclerosis

Furthermore, we explored the infiltration of immune cells in different samples. Both results of heatmaps (Fig. 6a, b) and violin plots (Fig. 6c, d) showed significant changes in a variety of immune cells in the periodontitis dataset GSE16134 and the MS dataset merged by GSE108000 and GSE135511, especially T cells and B cells. Additionally, analysis of the correlation between immune cells and candidate biomarkers revealed a positive association between regulatory T cells, natural killer cells, mast cells, immature dendritic cells and gamma delta T cells with *CFI* in both periodontitis samples and MS samples. In MS and periodontitis samples, there was a positive correlation between immature dendritic cells and *DDIT4L*. In samples with periodontitis and MS, type 1 T helper cells, T follicular helper cells, regulatory T cells plasmacytoid dendritic cells, natural killer T cells, natural killer cells, MDSCs, mast cells, macrophage, immature B cells, gamma delta T cells, activated B cells, activated dendritic cells, activated CD4 T cells and activated CD8 T cells showed a positive correlation with *FAM46C* (Fig. 6e, f).

### *CFI*, *DDIT4L* and *F4AM6C* were upregulated in patients with periodontitis and MS compared with healthy controls

**Fig. 7:**
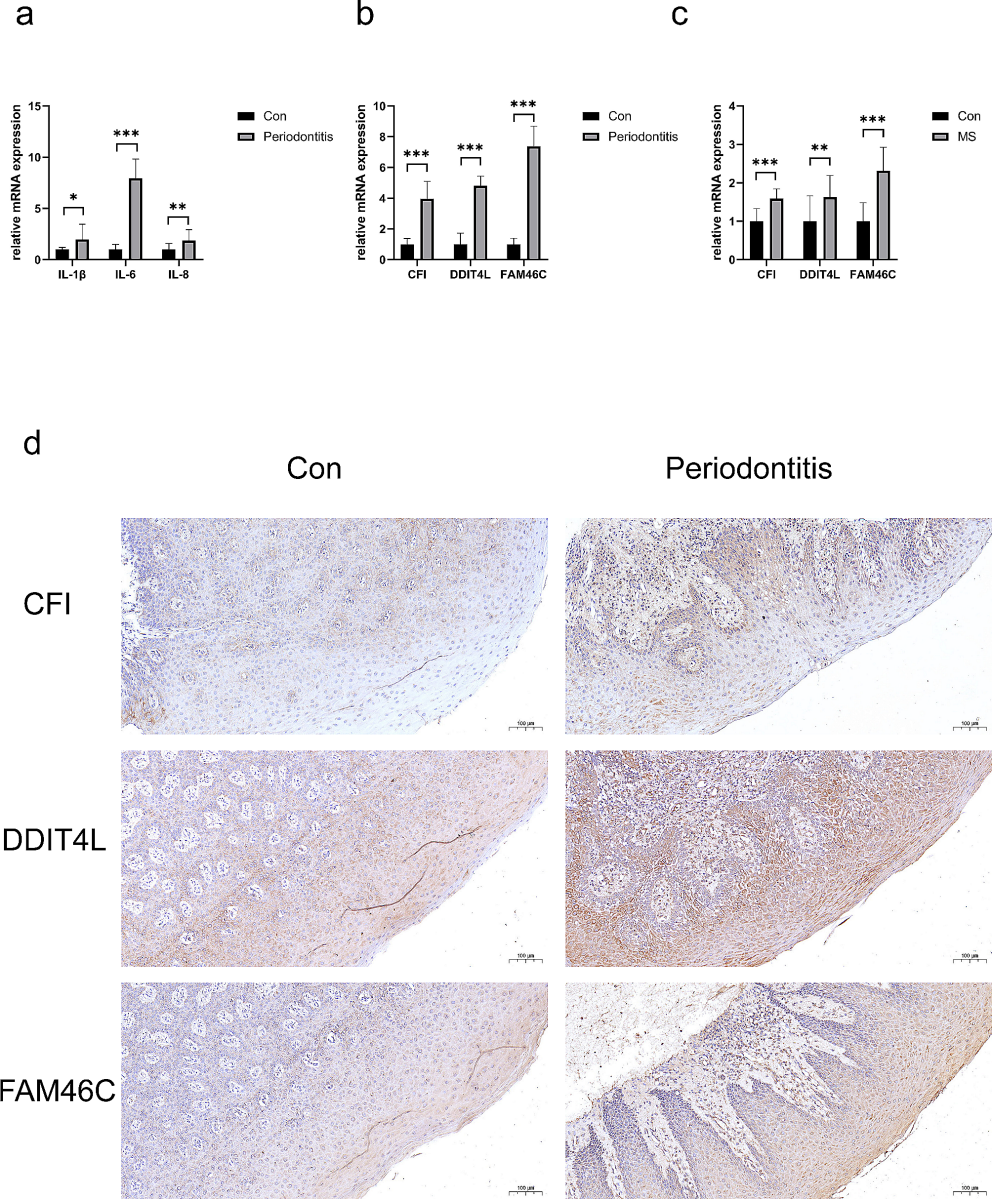
*CFI*, *DDIT4L* and *FAM46C* was upregulated in patients with periodontitis and MS compared with healthy controls. (a) qRT-PCR results show the mRNA expression of IL-1β, IL-6 and IL-8 in the gingivae of healthy and periodontitis (n_con_=5, n_case_=5). GAPDH was used for normalization relative to the control group. (b) qRT-PCR results show the mRNA expression of *CFI*, *DDIT4L* and *FAM46C* in the gingivae of healthy and periodontitis (n_con_=5, n_case_=5). GAPDH was used for normalization relative to the control group. (c) qRT-PCR results show the mRNA expression of *CFI*, *DDIT4L* and *FAM46C* in the peripheral blood of healthy and MS (n_con_=10, n_case_=5). GAPDH was used for normalization relative to the control group. (d) Immunohistochemistry staining of *CFI*, *DDIT4L* and *FAM46C* in the gingivae of healthy and periodontitis. Con, control; MS: Multiple sclerosis

To further validate the diagnostic values of three candidate markers, qPCR and immunohistochemical staining were used to verify their expressions in periodontitis and MS samples. qRT-PCR results indicated that mRNA levels of the pro-inflammatory cytokines (IL-1, IL-6, and IL-8) (Fig. 7a) and also *CFI*, *DDIT4L*, *F4AM6C* (Fig. 7b) were upregulated in patients with periodontitis compared with healthy controls. Similarly, qRT-PCR results (Fig. 7c) indicated that the mRNA levels of the *CFI*, *DDIT4L*, and *F4AM6C* were upregulated in patients with MS compared with healthy controls. Results of immunohistochemical staining revealed that *CFI*, *DDIT4L*, and *FAM46C* were upregulated in periodontitis samples compared with healthy controls (Fig. 7d).

## Discussion

Periodontitis, a chronic inflammatory disease, causes systemic inflammation and contributes to the development of several neurodegenerative diseases, such as MS [8, 13]. However, the mechanisms remain to be revealed. Additionally, the lack of sufficient knowledge regarding the pathogenesis of MS has impeded the progress of treatment options. Through the use of large-scale data, bioinformatics techniques offer a thorough knowledge of numerous illnesses at the molecular level [14, 15]. Moreover, it is also particularly important for identifying potential biomarkers for the diagnosis and prognosis of human diseases [16, 17]. Nevertheless, there were few reports on their utilization for screening potential biomarkers in patients with periodontitis combined with MS.

In this study, we used WGCNA to look into the common pathways by combining the transcriptomes of MS and periodontitis. Meanwhile, we uncovered possible intersecting genes, common pathways, and infiltration of immune cells between periodontitis and MS through multiple methods. Results of our study discovered that the most significant crosstalk genes between periodontitis and MS were *FAM46C*, *SLC7A7*, *LY96*, *CFI*, *DDIT4L*, *CD14*, and *IGJ*, which may be associated with response to molecules of bacterial origin. Then, it was discovered that *CFI*, *DDIT4L*, and *FAM46C* are useful diagnostic markers for periodontitis and MS. T cells and B cells are essential in developing MS and periodontitis, according to the results of immune infiltration.

The findings of this research imply that the primary genes involved in the cross-talk between MS and periodontitis are linked to a bacterial molecular response. As we all know, periodontitis is an inflammatory disease, and bacteria play an important role in its pathogenesis [18]. Studies have demonstrated that the pathogens of periodontitis include a variety of bacteria, such as *Actinomyces aggregator, P. gingivalis, Forsetana, Treponema dentalis*, and *Clostridium nucleatus*. These bacteria can cause gingival cell death and periodontal tissue damage by secreting lipopolysaccharide (LPS) and a variety of toxic substances, producing a variety of inflammatory factors. These cytokines can also spread through the blood, causing a systemic inflammatory response that triggers MS [8]. In addition to being transmitted through the blood, some bacteria can directly stimulate nerve immune cells to activate an inflammatory response. For instance, glial cells, the main immune cells in the nervous system, have been discovered to be stimulated by *P. gingivalis* and its products lipopolysaccharide to produce pro-inflammatory mediators such as nitric oxide (NO) and prostaglandin E2 (PGE2), leading to demyelination and aggravating MS [19]. These results imply that bacterial factors are critical in developing MS and periodontitis and may account for part of the greater incidence of MS in patients with periodontitis. The KEGG enrichment analysis revealed that these crosstalk genes are involved in ALD, the complement and clotting cascade, NF-κB signaling pathway, and Toll-like receptor signaling pathways. Studies have indicated that *P. gingivalis* can worsen ALD by changing the composition of intestinal microbiota and the immune response of the host [20]. Moreover, ALD has an increased risk of MS development [21]. Meanwhile, the involvement of complement and coagulation cascade in the mechanisms of periodontitis and MS has been demonstrated [22–24]. NF-κB is a signaling pathway that plays a crucial role in regulating immune and inflammatory responses. Activation of NF-κB signaling pathway can enhance osteoclast differentiation and exacerbate periodontitis by increasing the expression of IL-1β and various inflammatory factors [25, 26]. Furthermore, activation of NF-κB signaling pathway can also impact MS by stimulating peripheral immunity and inflammatory responses in the central nervous system [27]. Additionally, Toll-like receptor signaling pathways have also been shown to mediate the development of periodontitis and MS by regulating immune responses [28, 29].

This study explored the potential immunological connection between MS and periodontitis in the preliminary stages. According to our findings, the immunological patterns of the MS and periodontitis groups were considerably different from those of the control group, with the increase in B cells and T cells being particularly noticeable. Multiple infections invading the host and setting off an immune response cause periodontitis. *P. gingivalis*, the main pathogenic bacterium responsible for periodontitis, has been identified to release a variety of virulence factors, which in turn trigger the production of pro-inflammatory molecules, leading to an increase in the number of local B cells and T cells. Peripherally activated T-cell and B-cell interactions additionally trigger MS. It is generally known that B cells play important roles in the development of MS. For instance, B cells in MS patients may emit not only antibodies but also soluble toxic substances that, by their proliferation, harm oligodendrocytes and neurons [30]. Meanwhile, many B-cell subtypes, including memory B-cells and plasma mother cells, have been observed in the cerebrospinal fluid (CSF) of MS patients, especially memory B-cells and plasma mother cells [31]. More importantly, the success of treating MS by depleting B cells using anti-CD20 antibodies strongly highlights the importance of B cells in MS [30]. Moreover, studies have shown that CD4 T lymphocytes, particularly helper T cells 1 (Th1) and 17 (Th17), can pass the blood-brain barrier in response to myelin antigens, infiltrate the central nervous system, and trigger inflammation. Among them, Th1 and Th17 can aggravate MS by secreting IFN-γ and IL-17 [32]. It’s interesting to note that one study discovered that *P. gingivalis* infection can boost the impact of T lymphocytes on CNS autoantigens [19]. Therefore, periodontal disease may exacerbate MS by increasing the sensitivity of T and B cells to autoimmune antigens.

To improve the accuracy of testing biomarkers, we choose datasets with large sample sizes as much as possible. In our research, the periodontitis dataset GSE16134 contained 310 samples of gingival tissue, while the MS dataset, which was created by merging GSE108000 and GSE135511, contained 90 samples of brain tissue. The receiver operator curve (AUC) is employed to evaluate the diagnostic efficacy of biomarkers. ROC curve showed that the AUC values of *CFI*, *DDIT4L*, and *FAM46C* in the diagnosis of periodontitis were 0.830, 0.795, and 0.896, while the AUC values in the diagnosis of MS were 0.775, 0.820, and 0.946. These results suggest that *CFI*, *DDIT4L*, and *FAM46C* have a high capacity to predict periodontitis and MS.

*Family with sequence similarity 46 member C* (*FAM46C*), a non-standard poly(A) polymerase, was found to be a significant crosstalk gene between periodontitis and MS. Previous evidence has shown that *FAM46C* can inhibit tumor growth through a variety of pathways [33]. In addition, emerging evidence has shown that *FAM46C* can regulate immune responses. M1/M2 imbalance is one of the manifestations of periodontitis and MS [34, 35]. Studies have found that *FAM46C* can promote the polarization of M2 and alleviate the immune response [36]. This may be one of the mechanisms by which *FAM46C* participates in periodontitis and MS. The results of the ssGSEA study showed that *FAM46C* was significantly positively associated with macrophages in periodontitis and MS samples, which also jointly emphasized the involvement of *FAM46C* in these two diseases of pathology through a mediated immune response.

*DNA-damage-inducible transcript 4* (*DDIT4L*) was found to be a gene that regulates autophagy and promotes autophagy by inhibiting the mTOR signaling pathway [37]. As we know, autophagy plays a significant part in innate immunity and has been linked to many inflammatory diseases [38]. In the pathogenesis of periodontitis, autophagy has been discovered to activate and regulate inflammation by promoting or inhibiting cytokines and lead to bone loss by disrupting the balance between osteogenesis and osteolysis [39, 40]. In addition, studies have shown that autophagy has a dual function in MS. On the one hand, myelin antigen presentation by CD4 T cells can be enhanced by enhancing the process of autophagy, thus aggravating MS. On the other hand, defective autophagy leads to abnormal clearance of inflammatory bodies and myelin debris in microglia and promotes pro-inflammatory phenotypes [31]. The above evidence indicates that *DDIT4L* may play a role in periodontitis-mediated MS by regulating autophagy. However, further experiments are needed to confirm this speculation.

*Complement Factor I* (*CFI*), a family of soluble serine proteases, can regulate the complement system by inactivating C3b and C4b [41]. However, less research has been reported on *CFI* in periodontitis and MS, and the evidence below suggests that *CFI* may participate in both diseases by regulating the complement system. Accumulated evidence has demonstrated that the complement system is implicated in multiple neurodegenerative diseases. It has been shown that the complement system is activated at the onset of MS, and the expression levels of C3 and C4 are increased [42]. In addition, the accumulation of C3b can cause damage to neurons through the activation of C5a [43]. The expression of C3, C3b, and C4b was also discovered to be elevated in the gingival tissue of individuals with periodontitis, and its expression was found to be positively connected with the severity of the condition. Meanwhile, using C3b/C4b inhibitors can alleviate alveolar bone loss in periodontitis [44]. These findings suggest that *CFI* may influence periodontitis-mediated MS by regulating the transformation of C3b and C4b.

In summary, our study revealed a correlation between periodontitis and MS using bioinformatic analyses, suggesting that MS can be prevented by improving oral hygiene and treating periodontitis, and providing guidance for the treatment of patients with periodontitis combined with MS. More importantly, *FAM46C*, *SLC7A7*, *LY96*, *CFI*, *DDIT4L*, *CD14*, *C5AR1* and *IGJ* were the most significant crosstalk genes between periodontitis and MS, and *CFI*, *DDIT4L*, *FAM46C* can be used as potential biomarkers for the diagnosis of periodontitis and MS. Immune responses driven by B cells and T cells are crucial in the pathogenesis of periodontitis and MS.

### Electronic supplementary material

Below is the link to the electronic supplementary material.

Supplementary Material 1

Supplementary Material 2

## Data Availability

Publicly available datasets were analyzed in this study. This data can be found at GEO data repository (https://www.ncbi.nlm.nih.gov/geo/) and include the accession numbers: GSE16134, GSE108000, GSE135511, GSE1334 and GSE38010.
