# Supplementary material for: Exploration of potential shared gene signatures between periodontitis and multiple sclerosis
Source: BMC Oral Health. 2024 Jan 13;24:75. doi: 10.1186/s12903-023-03846-7 (PMC10788039; doi:10.1186/s12903-023-03846-7)
Supplement: Supplementary file 1 — Supplementary Material 1 [file 12903_2023_3846_MOESM1_ESM.docx]

| Gene | Forward 5’-3’ | Reverse 5’-3’ |
| --- | --- | --- |
| GAPDH | CTTTGGTATCGTGGAAGGACTC | GTAGAGGCAGGGATGATGTTCT |
| CFI | CTCAGCAGAGACAAAGAC | GTGGAAGCACAGAAATAAC |
| DDIT4L | TGCTGGACTGTGGCTATCAC | GAGGTTGGGTTCAGGAACAA |
| FAM46C | CCGCCGTATAAGAACGGAG | AGAAGAGGAGGCGACAGAG |
| IL-1β | AGAAGTACCTGAGCTCGCCA | CTGGAAGGAGCACTTCATCTGT |
| IL-6 | AGGCACTGGCAGAAAACAAC | TTTTCACCAGGCAAGTCTCC |
| IL-8 | GTGTGAAGGTGCAGTTTTGC | TGTGGTCCACTCTCAATCACTC |

**Table S1.** Sequences of the primers used for RT-qPCR.
