# Supplementary material for: Exploration of potential shared gene signatures between periodontitis and multiple sclerosis
Source: BMC Oral Health. 2024 Jan 13;24:75. doi: 10.1186/s12903-023-03846-7 (PMC10788039; doi:10.1186/s12903-023-03846-7)
Supplement: Supplementary file 2 — Supplementary Material 2 [file 12903_2023_3846_MOESM2_ESM.docx]

**Supplemental Fig. S1**

a


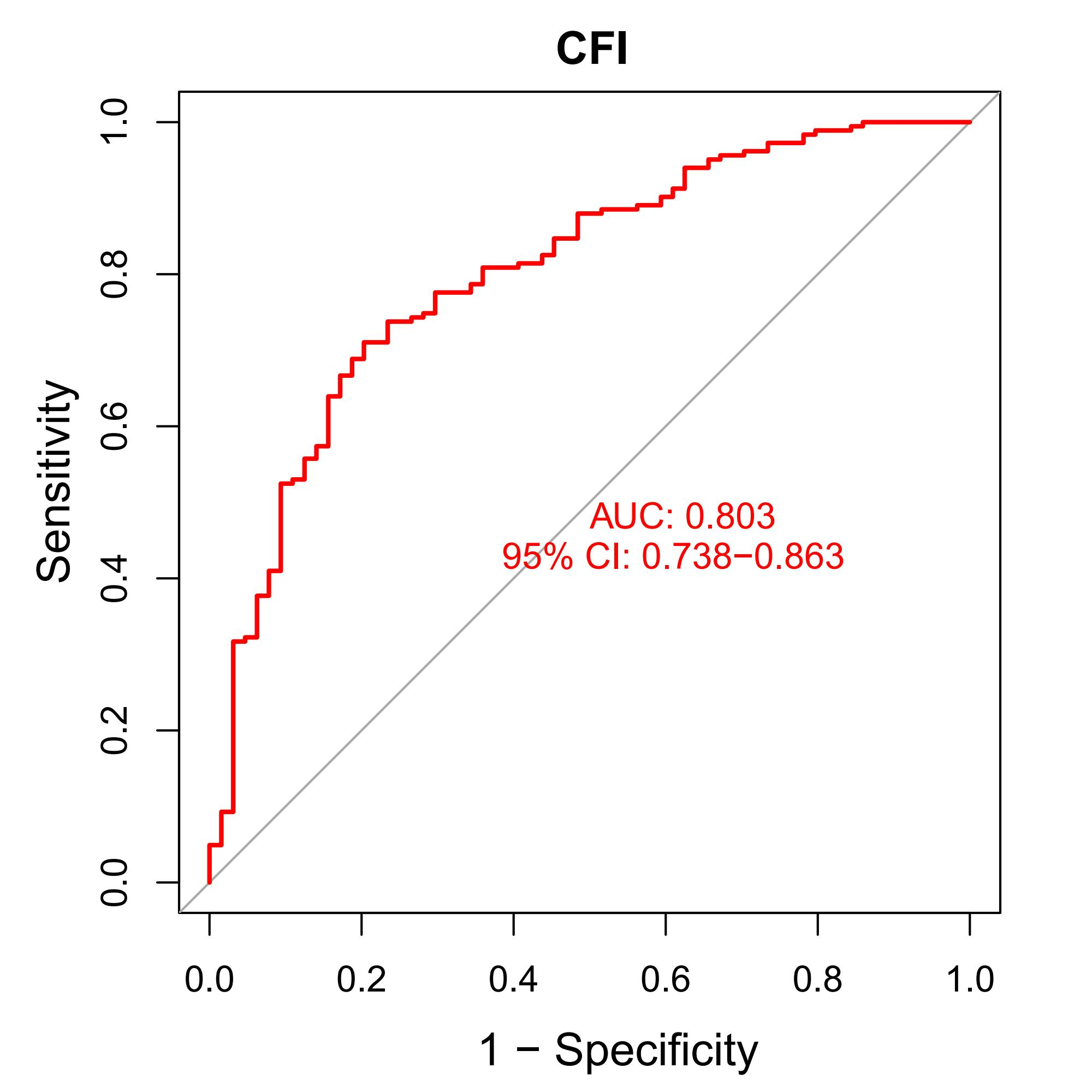

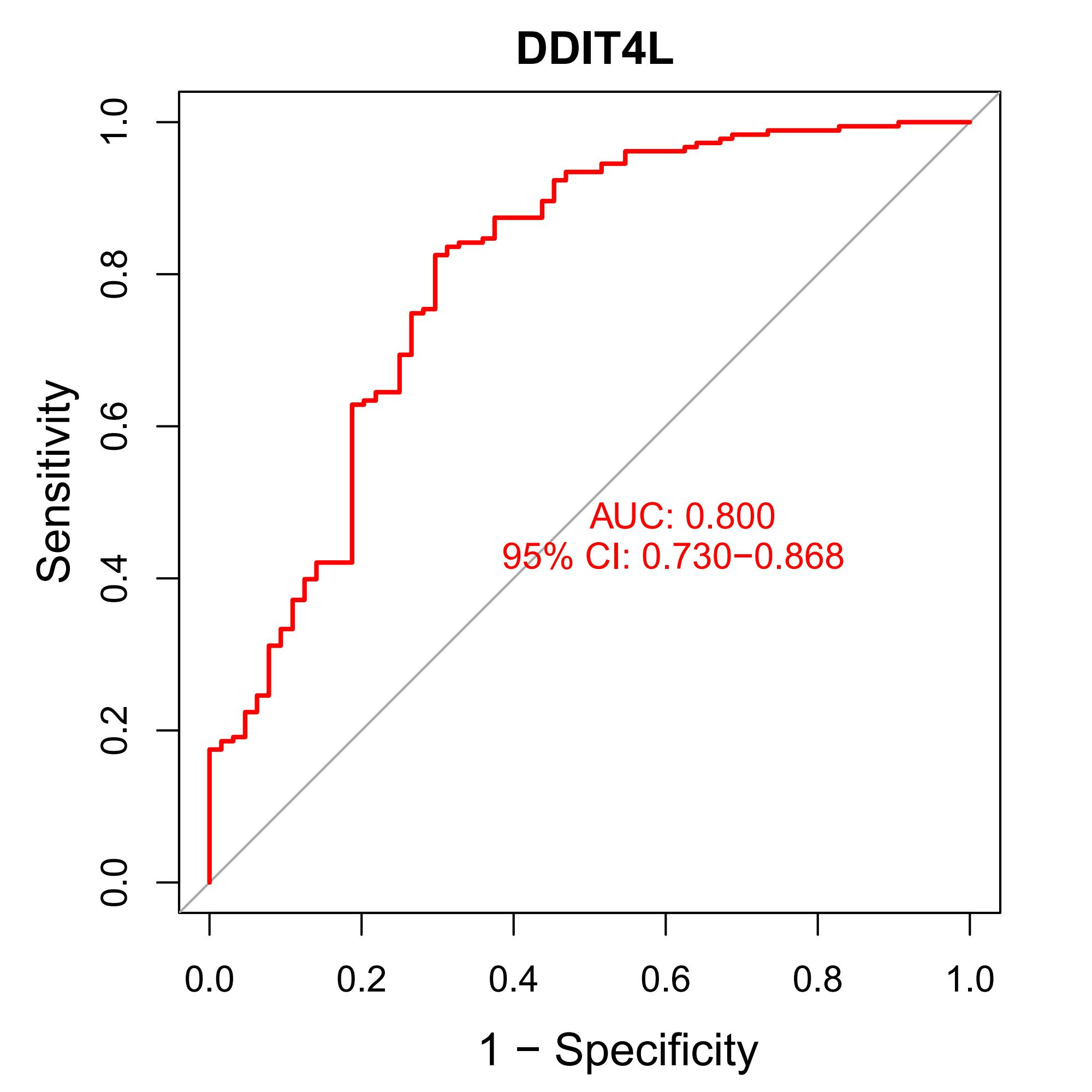

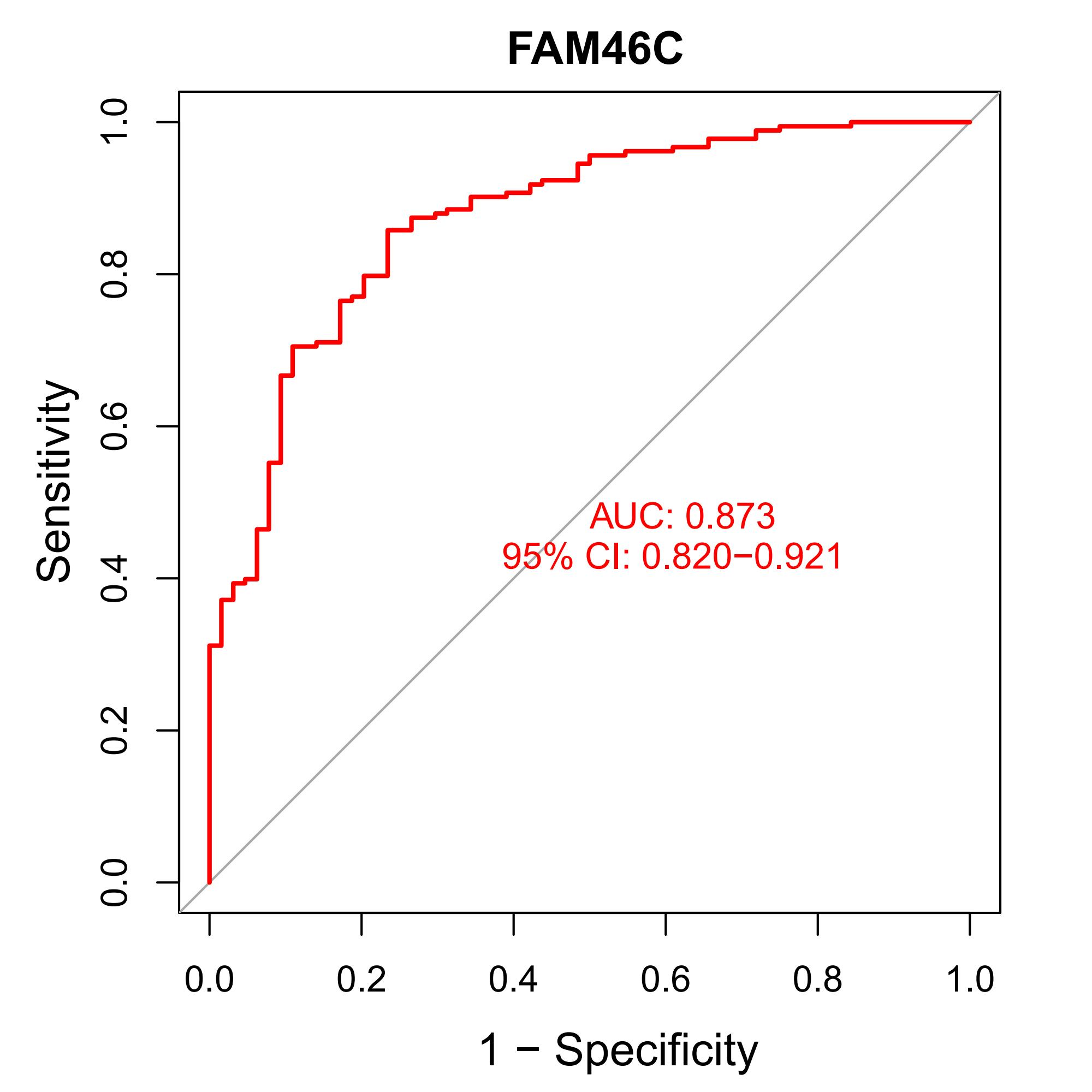

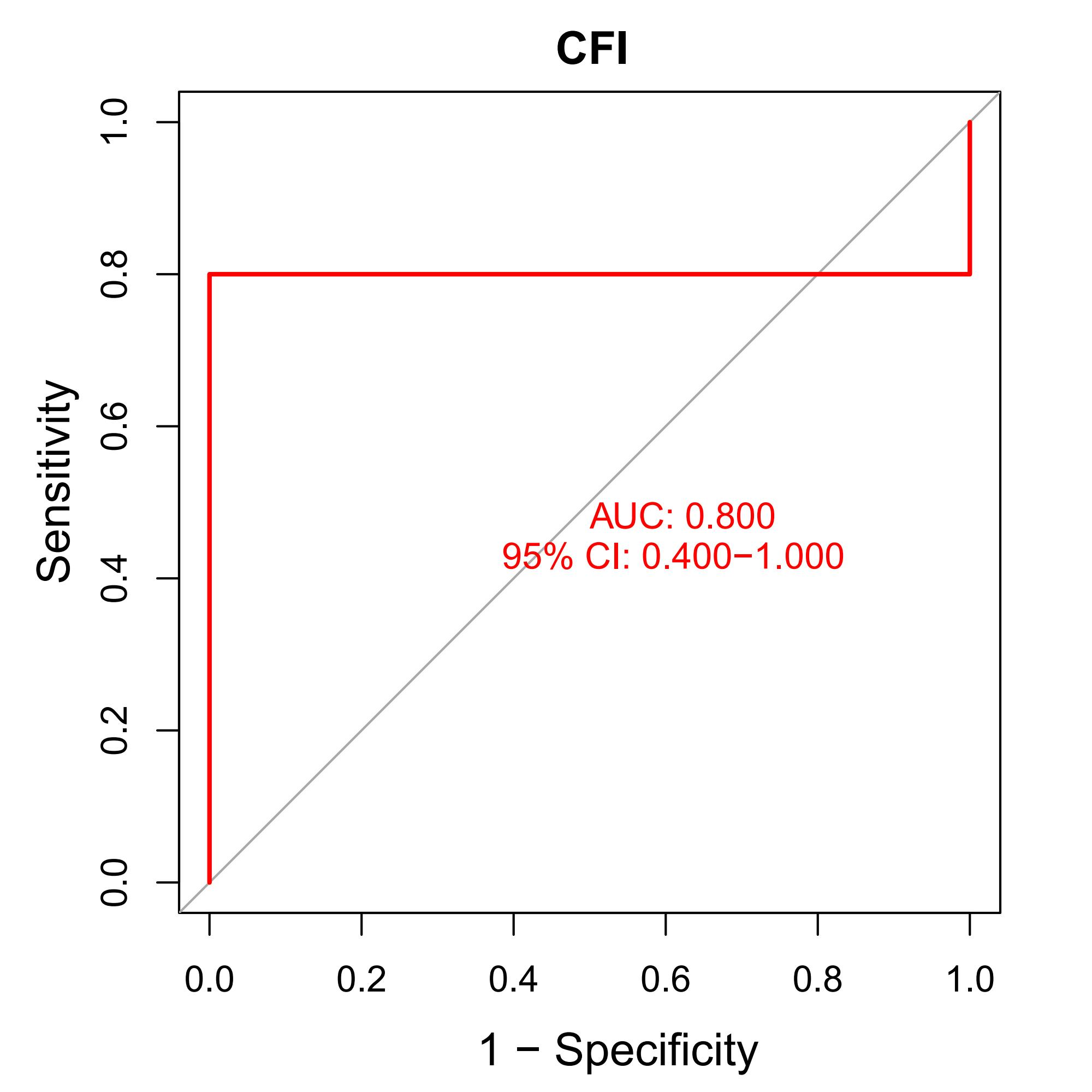

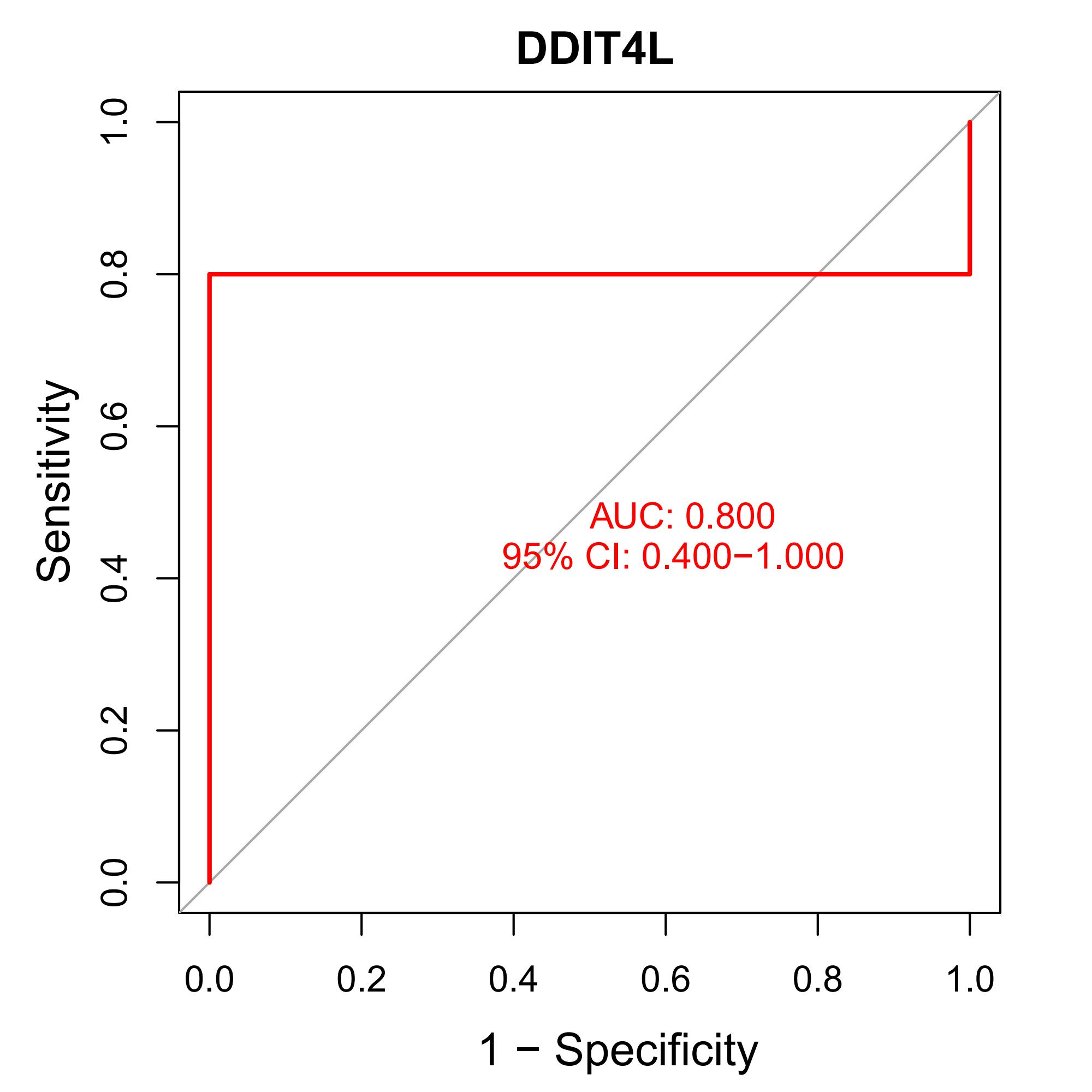

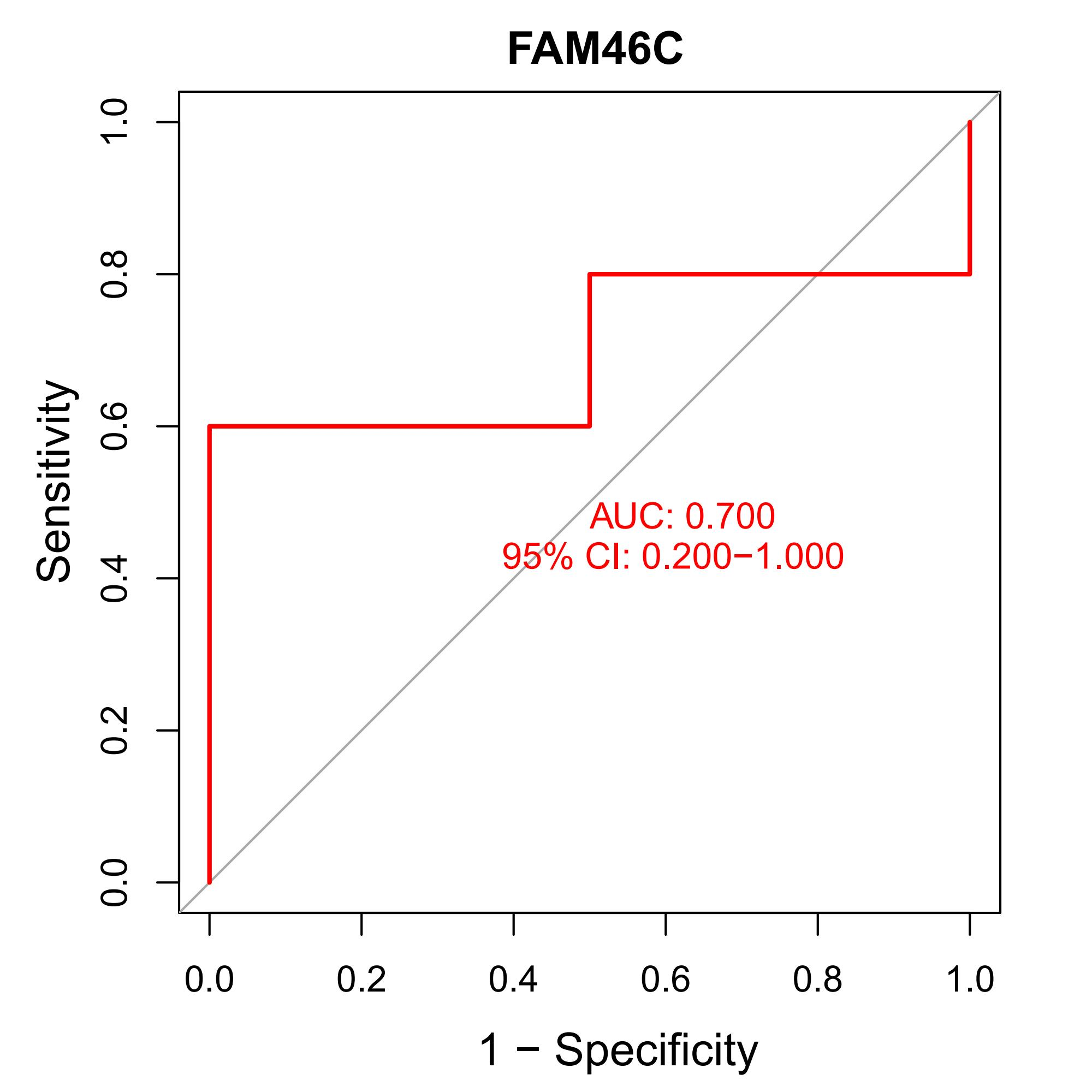


b

Fig. S1. Validation of potential shared diagnostic genes in external periodontitis and MS datasets. **(a)** Expression of CFI, DDIT4L and FAM46C in the periodontitis database GSE10334. **(b)** Expression of CFI, DDIT4L and FAM46C in the periodontitis database in the MS database GSE38010. Con: control; MS: Multiple sclerosis. *P < 0.05; **P < 0.01; ***P < 0.001.
